# Obesity-dependent increase in RalA activity disrupts mitochondrial dynamics in white adipocytes

**DOI:** 10.21203/rs.3.rs-2923510/v1

**Published:** 2023-06-02

**Authors:** Wenmin xia, Preethi Veeragandham, Yu Cao, Yayun Xu, Torrey Rhyne, Jiaxin Qian, Chao-Wei Hung, Peng Zhao, Ying Jones, Hui Gao, Christopher Liddle, Ruth Yu, Michael Downes, Ronald Evans, Mikael Ryden, Martin Wabitsch, Shannon Reilly, Jianfeng Huang, Alan Saltiel

**Affiliations:** University of California San Diego; University of California San Diego; University of California San Diego; University of California San Diego; University of California San Diego; University of California San Diego; University of North Carolina; University of Texas Health Science Center at San Antonio; University of California San Diego; Karolinska Institutet; Salk Institute for Biological Studies; Salk Institute; Salk Institute; Salk Institute for Biological Studies; Karolinska Institute; Ulm University Medical Center; Weill Medical College of Cornell University; Salk Institute for Biological Studies; UCSD

## Abstract

Mitochondrial dysfunction is a characteristic trait of human and rodent obesity, insulin resistance, and fatty liver disease. Here we report that mitochondria undergo fragmentation and reduced oxidative capacity specifically in inguinal white adipose tissue after feeding mice high fat diet (HFD) by a process dependent on the small GTPase RalA. RalA expression and activity are increased in white adipocytes from mice fed HFD. Targeted deletion of *Rala* in white adipocytes prevents the obesity-induced fragmentation of mitochondria and produces mice resistant to HFD-induced weight gain via increased fatty acid oxidation. As a result, these mice also exhibit improved glucose tolerance and liver function. *In vitro* mechanistic studies revealed that RalA suppresses mitochondrial oxidative function in adipocytes by increasing fission through reversing the protein kinase A-catalyzed inhibitory Ser^637^phosphorylation of the mitochondrial fission protein Drp1. Active RalA recruits protein phosphatase 2A (PP2Aa) to specifically dephosphorylate this inhibitory site on Drp1, activating the protein, thus increasing mitochondrial fission. Adipose tissue expression of the human homolog of Drp1, *DNML1,* is positively correlated with obesity and insulin resistance in patients. Thus, chronic activation of RalA plays a key role in repressing energy expenditure in obese adipose tissue by shifting the balance of mitochondrial dynamics towards excessive fission, contributing to weight gain and related metabolic dysfunction.

## Summary

Obesity has become a worldwide epidemic ^1^, dramatically increasing the incidence of type 2 diabetes, nonalcoholic steatohepatitis, and other cardiometabolic abnormalities ^2–4^. During the development of obesity, white adipose tissue (WAT) chronically expands and undergoes metabolic changes characterized by hormone insensitivity, inflammation, fibrosis, and apoptosis ^5,6^. While mitochondria play an important metabolic role in healthy adipocytes, oxidizing fuel to produce ATR and generating heat during thermogenesis, mitochondrial function is impaired in obese subjects ^7–10^. However, what drives mitochondrial damage, and how it contributes to obesity and its many complications remain unknown.

Obesity is associated with hyperinsulinemia and diabetes ^11,12^ and studies have suggested a link between mitochondrial dysfunction, reduced energy expenditure and insulin resistance ^13^. Altered mitochondrial oxidative function has been observed in muscle as well as adipose tissue from obese compared to normal-weight individuals ^14–18^, and adipocytes from obese subjects contain fewer mitochondria compared to lean counterparts ^15^. Moreover, mitochondria in muscle of obese subjects are fragmented ^14^. Changes in mitochondrial size and number are controlled by the dynamic balance of fusion and fission ^19^. Fusion is crucial for the optimal control of mitochondrial number and integrity, particularly in response to changes in energy needs. Fission, which is catalyzed by the dynamin related protein Drp1, mediates mitochondrial division and quality control during cell division ^20^. However, mitochondrial fusion and fission are observed in many nondividing cells, indicating that the correct balance of these processes is crucial to adapting to energy needs and ensuring homeostasis.

Ral GTPases are members of the Ras superfamily involved in multiple cellular processes. We previously demonstrated that RalA is activated by insulin in adipocytes, and in turn interacts with members of the exocyst complex to target GLUT4 vesicles to the plasma membrane for docking and subsequent fusion, leading to increased glucose uptake ^21–23^. Insulin activates RalA through inhibitory phosphorylation of the RalGAP complex (RGC) ^24^, as well as localization of RGL2, a guanine-nucleotide exchange factor (GEF) for RalA ^25^. *In vivo* activation of RalA through targeted deletion of the RalGAP protein *Ralgapb* promotes glucose uptake into brown adipose tissue (BAT) ^26^, and dramatically improves glucose homeostasis in mice on HFD. Similarly, targeted deletion of *Ralgapa1* improves postprandial glucose and lipid disposal into muscle ^27^.

We report here that RalA gene and protein expression and activity are increased in adipocytes from obese mice, and further that targeted deletion of *Rala* in white, but not brown adipocytes, attenuates HFD-induced obesity, due to dramatically increased energy expenditure and mitochondrial oxidative phosphorylation, specifically in inguinal white adipose tissue (iWAT). These beneficial effects of RalA deletion were driven by a reversal of the increased mitochondrial fission in white adipocytes induced by feeding mice HFD. *In vitro* studies revealed that RalA interacts with PP2Aa to promote the dephosphorylation of inhibitory S637 on Drp1, rendering Drp1 active, leading to excessive fission and mitochondrial fragmentation. Taken together, these data reveal that persistent elevation of RalA in obesity produces mitochondrial dysfunction in white adipocytes, with profound effects on systemic metabolism.

## White adipocyte-specific Rala deletion protects mice from high fat diet-induced obesity

**Figure 1:**
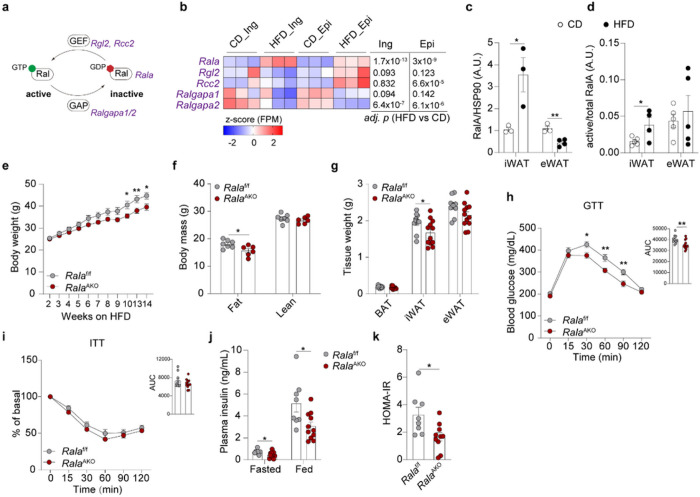
White adipocyte-specific *Rala* deletion protects mice from high fat diet-induced obesity. **a,** Scheme illustrating RalA activation network involving genes encoding RalA, GEF and GAP **b,** RNA-seq analysis of primary inguinal and epididymal mature adipocytes isolated from mice (n = 3) under 16-weeks HFD feeding. Heatmap displays transcriptional expression as z-scored FPM values. Adjusted p-values are indicated and considered significant with value < 0.05. **c,** Quantification of RalA protein content in mature adipocytes from iWAT and eWAT of mice fed with CD or HFD for 16 weeks (n = 3–4). **d,** Quantification of RalA GTPase activity in iWAT and eWAT of mice fed with CD or HFD for 4 weeks (n = 4). **e,** Body weight of *Rala*^f/f^ and *Rala*^AKO^ mice (n = 8–10) fed with 60% HFD. **f,** Body mass of *Rala*^f/f^ and *Rala*^AKO^ mice (n **=** 6–7) fed with HFD for 12 weeks, **g,** Fat depot weights of *Rala*^f/f^ and *Rala*^AKO^ mice (n = 10–12) fed with HFD for 12 weeks, **h,** GTT on 11-weeks HFD-fed *Rala*^f/f^ and *Rala*^AKO^ mice (n = 10–13); AUC was calculated from longitudinal chart, **i,** ITT on 12-week HFD-fed *Rala*^f/f^ and *Rala*^AKO^ mice (n = 11–12); AUC was calculated from longitudinal chart, **j,** Plasma insulin levels in 8-week HFD-fed *Rala*^f/f^ and *Rala*^AKO^ mice (n = 11). k, HOMA-IR was calculated using fasting glucose and insulin levels from 8-weeks HFD-fed *Rala*^f/f^ and *Rala*^AKO^ mice (n =8–10). The data **(c-k)** are shown as the mean ± SEM, *p < 0.05, **p < 0.01, ***p < 0.001 by unpaired t-test **(c, d, f, g, j, k)** or two-way ANOVA with Bonferroni’s multiple comparison as post-test **(e, h, i).**

RNA-seq analysis from isolated mature adipocytes derived from control and HFD-fed mice ^28^ revealed that *Rala* expression is significantly upregulated in adipocytes from eWAT and iWAT during obesity development, while *Ralgapa2* expression is downregulated (Fig. 1a,b). In addition, RalA protein content is increased in mature adipocytes from iWAT of obese mice (Fig. 1c, Extended Data Fig. 1a), accompanied by elevation of RalA-GTP binding (Fig. 1d, Extended Data Fig. 1b). We also observed a trend towards a positive correlation of the expression of the Ral GEF *RGL2* in adipose tissue with BMI in a large dataset of obese patients (Extended Data Fig. 1c,d). Together, these observations support the notion that adipocyte RalA activity is constitutively elevated in obesity.

To explore further whether RalA plays a role in glucose homeostasis and energy metabolism, we generated adipocyte-specific *Rala* knockout *(Rala*^AKO^) mice by crossing *Rala*-floxed mice with adiponectin-Cre transgenic mice. Compared to *Rala*^f/f^ littermates, *Rala*^AKO^ mice had a greater than 90% decrease of RalA protein in primary adipocytes from WAT and BAT, and an approximately 50% decrease in whole WAT, without changes in liver (Extended Data Fig. 1e). Insulin-stimulated GTP binding of RalA was diminished in WAT of *Rala*^AKO^ mice compared to control mice, and the same result was observed in primary adipocytes (Extended Data Fig. 1f).

We generated primary white adipocytes by differentiation of iWAT stromal vascular cells from control and KO mice. As previously seen in 3T3-L1 adipocytes ^22^ knockout of RalA completely prevented the translocation of GLUT4 from intracellular sites to the plasma membrane in response to insulin (Extended Data Fig. 1g). Moreover, insulin-stimulated glucose uptake in KO cells was significantly reduced in knockout cells (Extended Data Fig. 1h).

Adipocyte specific deletion of *Rala* had no effect on body weight in chow diet (CD)-fed mice, although these mice displayed a reduction in fat mass and depot weight (Extended Data Fig. 2a-c). Generally, adipocytes from iWAT were considerably smaller than those found in epididymal WAT (eWAT) from mice fed CD. Moreover, *Rala*^AKO^ mice had smaller adipocytes in iWAT compared to control mice fed with CD, while adipocytes were comparable in eWAT and BAT between the genotypes (Extended Data Fig. 2d). While *Rala*^AKO^ mice on chow diet showed no difference in glucose tolerance, there was a slight reduction in insulin tolerance when compared to *Rala*^f/f^ mice (Extended Data Fig. 2e,f). Insulin levels and HOMA-IR in *Rala*^AKO^ mice were not different from control mice fed with CD (Extended Data Fig. 2g,h). However, *Rala*^AKO^ mice gained significantly less weight than control littermates when challenged with 60% HFD (Fig. 1e). *Rala*^AKO^ mice showed a marked reduction of fat mass, with no change in lean body mass (Fig. 1f). Further analyses revealed that iWAT weight was significantly reduced in *Rala*^AKO^ mice, with no difference in eWAT and BAT (Fig. 1g). HFD increased adipocyte size in all fat depots, but the effect was most pronounced in iWAT; HFD-fed Rala^AKO^ mice displayed a trend towards smaller adipocytes in iWAT compared to control mice, but not in eWAT or BAT (Extended Data Fig. 2d). HFD-fed *Rala*^AKO^ mice exhibited a marked improvement in glucose tolerance compared to control mice, with no change in insulin tolerance (Fig. 1h,i), but with reduced insulin levels and improved HOMA-IR (Fig. 1j,k). Fasting glucose levels were comparable between the genotypes on either HFD or CD (Extended Data Fig. 2i,j).

To investigate further which adipose tissue depot is responsible for the reduced weight gain in *Rala*^AKO^ mice fed FIFD, we generated BAT-specific *Rala* knockout (*Rala*^BKO^) mice by crossing RalA-floxed mice with UCP1-Cre transgenic mice (Extended Data Fig. 2k). Although CD-fed *Rala*^BKO^ mice showed a reduction in BAT weight, presumably due to reduced glucose uptake, there were no differences in fat mass or depot weight compared with control mice (Extended Data Fig. 2l,m). Glucose and insulin tolerance tests (GTT and ITT) were identical between the genotypes on control diet (Extended Data Fig. 2n,o). Moreover, no differences in body weight, fat mass, tissue weight, GTT, or ITT were observed in HFD-fed *Rala*^BKO^ mice (Extended Data Fig. 2p-t). These results suggest that specific *Rala* deletion in WAT, especially in iWAT, protects mice against obesity.

## Loss of RalA in WAT ameliorates HFD-induced hepatic steatosis

**Figure 2:**
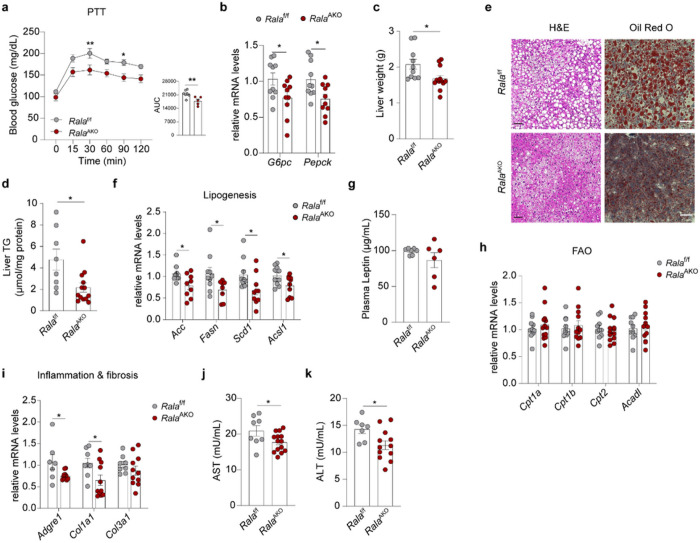
Loss of RalA in WAT ameliorates HFD-induced hepatic steatosis. **a,** Pyruvate tolerance test (PTT) was performed on overnight fasted *Rala*^**f/f**^ and *Rala*^**AKO**^ mice (n = 5–7) after 8 weeks of HFD feeding. AUC was calculated from PTT longitudinal chart, **b,** Relative mRNA expression of key gluconeogenic genes in livers of HFD-fed *Rala*^**f/f**^ and *Rala*^**AKO**^ mice (n = 10). **c,** Liver weight of HFD-fed *Rala*^**f/f**^ and *Rala*^**AKO**^ mice (n = 10–12). **d,** Triglyceride (TG) content in livers of HFD-fed *Rala*^**f/f**^ and *Rala*^**AKO**^ mice (n = 8–13). **e,** Representative H&E staining (left; n = 3) and Oil-Red-O staining (right; n = 3) of liver sections in HFD-fed *Rala*^**f/f**^ and *Rala*^**AKO**^ mice, scale bar =15 mm. **f,** Relative mRNA expression of lipogenic genes in livers of HFD-fed *Rala*^**f/f**^ and *Rala*^**AKO**^ mice (n **=** 9–10). **g,** Plasma leptin levels in HFD-fed *Rala*^**f/f**^ and *Rala*^**AKO**^ mice (n = 6–7). **h,** Relative mRNA expression of fatty acid oxidation (FAO) related genes in livers of HFD-fed *Rala*^**f/f**^ and *Rala*^**AKO**^ mice (n = 10–11). **i,** Relative mRNA expression of genes related to inflammation and fibrosis in livers of HFD-fed *Rala*^**f/f**^ and *Rala*^**AKO**^ mice (n = 7–11). **j, k,** Plasma AST **(j)** and ALT **(k)** activities in HFD-fed *Rala*^**f/f**^ and *Rala*^**AKO**^ mice (n = 7–14). The data **(a-d, f-k)** are shown as the mean ± SEM, *p < 0.05, **p < 0.01 by unpaired t-test **(b-d, f-k)** or two-way ANOVA with Bonferroni’s multiple comparison as post-test **(a).**

Since HFD-fed *Rala*^AKO^ mice showed an improved GTT without markedly altering insulin tolerance, we speculated that the improved glucose handling is due to reduced hepatic glucose production. To test this assumption, we performed a pyruvate tolerance test (PTT) in HFD-fed *Rala*^f/f^ and *Rala*^AKO^ mice. *Rala*^AKO^ mice exhibited substantially lower glucose excursions following pyruvate challenge compared to control mice (Fig. 2a). There was a significant downregulation of the hepatic gluconeogenic genes *G6pc* and *Pepck*(Fig. 2b). These data suggest that adipose tissue-specific *Rala* deletion improved glucose homeostasis partially through reduced hepatic glucose production.

Liver weights and triglyceride (TG) content were significantly reduced in HFD-fed *Rala*^AKO^ mice when compared to control mice (Fig. 2c,d). Both H&E and Oil-Red-O staining indicated less lipid accumulation in the liver of *Rala*^AKO^ mice (Fig. 2e). In line with histology results, lipogenic genes *(Acc, Fasn, Scd1 and Acsl1)* were expressed at significantly lower levels in the liver of *Rala*^AKO^ mice (Fig. 2f). However, plasma leptin levels (Fig. 2g) and hepatic expression of genes related to fatty acid oxidation (FAO) (Fig. 2h) were unchanged in *Rala*^AKO^ mice. In addition, inflammatory (*Adgre1*) and fibrosis-related (*Col1a1* and *Col3a1)* genes were expressed at lower levels in livers of *Rala*^AKO^ mice (Fig. 2i), as were aspartate aminotransferase (AST) and aminotransferase (ALT) activities (Fig. 2j,k). Of note, we did not observe a difference in liver weights in *Rala*^BKO^ compared to controls fed with HFD (Extended Data Fig. 2r). Together, these observations suggest that WAT-specific deletion of *Rala* systemically regulates lipid metabolism to ameliorate liver steatosis and damage in obesity.

## RalA deficiency in WAT increases energy expenditure and mitochondrial oxidative phosphorylation

**Figure 3:**
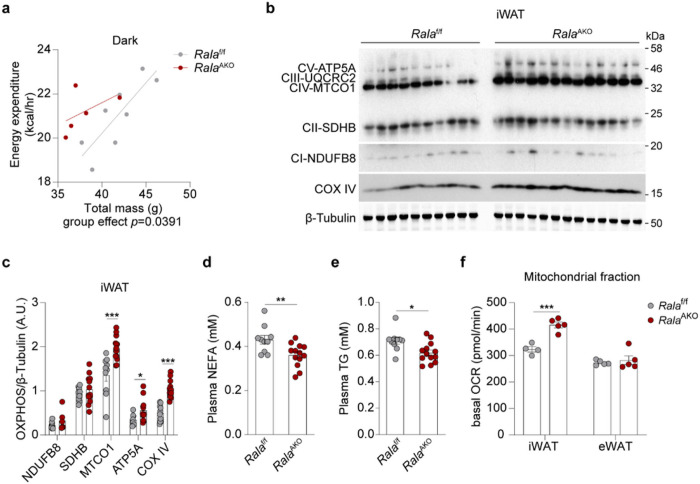
RalA deficiency in WAT increases energy expenditure and mitochondrial oxidative phosphorylation. **a,** Regression plot of energy expenditure (EE) measured in HFD-fed *Rala*^f/f^ and *Rala*^AKO^ mice (n = 5–8) during dark phase. ANCOVA test was performed using body weight (BW) as a covariate, group effect *p* = 0.0391. **b, c**, Immunoblot (**b**) and quantification (**c**) of OXPHOS proteins in iWAT of HFD mice (n = 10–13). **d, e,** Plasma non-esterified fatty acid (NEFA, **d**) and TG (**e**) levels in HFD-fed *Rala*^f/f^ and *Rala*^AKO^ mice (n = 10–13). **f**, Basal oxygen consumption rate (OCR) in mitochondria measured by Seahorse. Mitochondrial fractions were isolated from primary mature adipocytes in iWAT or eWAT of HFD-fed *Rala*^f/f^ and *Rala*^AKO^ mice (n = 4–5). The data (**c-f**) are shown as the mean ± SEM, *p < 0.05, **p < 0.01,***p < 0.001 by unpaired t-test (**c-f**).

To explore why adipose tissue *Rala* deletion protects mice from HFD-induced hepatic steatosis, weight gain, and glucose intolerance, we investigated energy metabolism in *Rala*^AKO^ mice with metabolic cage studies. While *Rala* ablation in adipocytes did not affect energy metabolism and food intake in mice fed CD (Extended Data Fig. 3a-e), HFD-fed *Rala*^AKO^ mice displayed a significant increase in energy expenditure (EE) during the dark phase as determined by ANCOVA using body weight as a covariate (Fig. 3a). Concordantly, oxygen consumption in *Rala*^AKO^ mice was similarly increased compared to controls (Extended Data Fig. 3f), although there was no difference in respiratory exchange rate (RER), locomotor activity, or food intake between the genotypes (Extended Data Fig. 3g-i). In contrast, *Rala*^BKO^ mice fed either control or HFD were identical to control littermates in EE, 0_2_ consumption, RER, locomotor activity, and food intake (Extended Data Fig. 3j-n). These observations demonstrate that *Rala* deficiency specifically in WAT increases energy expenditure.

Increased energy expenditure is an indirect reflection of increased mitochondrial oxidative activity. Thus, we assessed the expression of mitochondrial proteins in fat depots. Oxidative phosphorylation (OXPHOS) proteins were markedly increased in iWAT of *Rala*^AKO^ mice (Fig. 3b,c), but not in eWAT (Extended Data Fig. 3o,p). Complex I and Complex II levels were modestly increased in BAT of *Rala*^AKO^ mice (Extended Data Fig. 3q,r). This may occur because of systemic metabolic improvement in *Rala*^AKO^ mice rather than a cell-autonomous BAT function, since HFD-fed *Rala*^BK0^ mice did not show an improved metabolic phenotype. In this regard, plasma FFA and TG levels in HFD-fed *Rala*^AKO^ mice were significantly lower (Fig. 3d,e). To test the possible involvement of a generalized browning of iWAT, we also examined thermogenic markers. *Ucp1, Cidea,* and *Prdm16* expression was identical between the genotypes in all three fat depots, indicating that the improvement in energy expenditure in *Rala*^AKO^ mice did not reflect the development of beige adipose tissue (Extended Data Fig. 3s).

### RalA knockout in white adipocytes increases mitochondrial activity and fatty acid oxidation

**Figure 4:**
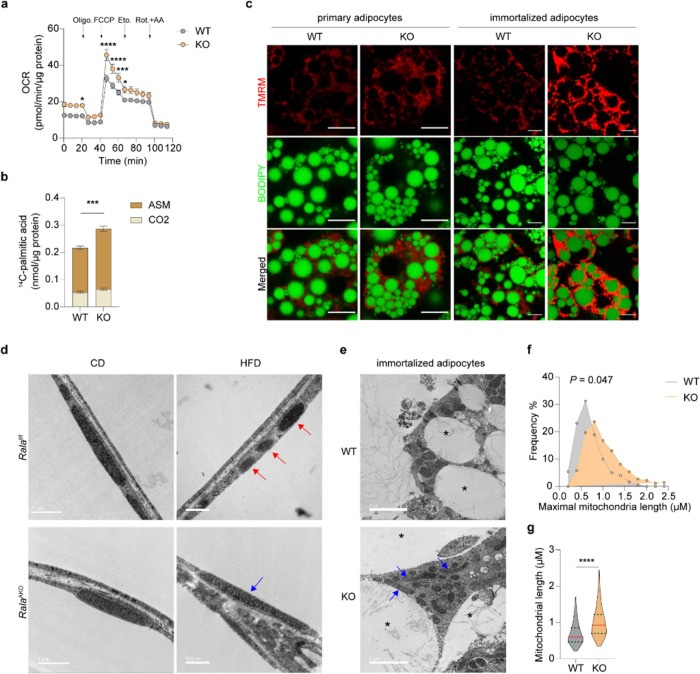
*RalA* knockout in white adipocytes increases mitochondrial activity and fatty acid oxidation via preventing obesity-induced mitochondrial fission in iWAT. **a,** OCR was measured in differentiated primary adipocytes (n = 8). Vertical arrows indicate injection ports of indicated chemicals, **b,** ^**14**^C-palmitic acid oxidation in differentiated primary adipocytes under basal condition (n = 3–4). ASM: acid-soluble metabolites, **c,** Representative confocal images (n = 3) of live primary and immortalized adipocytes stained with TMRM (red) and BODIPY (green). Scale bar = 15 μm. **d,** Representative electron microscope (EM) images of iWAT from CD-fed and HFD-fed *Rala*^**f/f**^ and *Rala*^**AKO**^ mice (n = 3). Red arrow indicates fissed mitochondria; blue arrow indicates elongated mitochondria. Scale bar = 1 μm (CD) or = 500 nm (HFD). **e,** Representative EM images of WT and *RalA* KO immortalized adipocytes (n = 3). blue arrow indicates elongated mitochondria; asterisk indicates lipid droplet. Scale bar = 2 μm. **f, g,** Histogram **(f)** and violin plot **(g)** of maximal mitochondrial length in immortalized adipocytes (n = 100–180). Violin plot is presented as violin: 25^**th**^ to 75^**th**^ percentile, and whiskers: min to max. The data **(a, b)** are shown as the mean ± SEM, *p < 0.05, **p < 0.01, ***p < 0.001, ****p < 0.0001 by unpaired t-test **(b, g),** two-way ANOVA alone **(f)** or with Bonferroni’s multiple comparison as post-test **(a).**

We sought to evaluate further the mechanisms underlying improved energy metabolism in *Rala*^AKO^ mice, and directly assessed mitochondrial activity in adipocytes. Measurements of basal respiration revealed that oxygen consumption rate (OCR) was significantly increased in mitochondria isolated from KO iWAT compared to that from control mice, but was similar in eWAT mitochondria of *Rala*^f/f^ and *Rala*^AKO^ mice (Fig. 3f). We also noted that both basal and maximal respiration were significantly higher in primary differentiated adipocytes from KO mice, and the difference in maximal respiration was blunted by the addition of the CPT1 inhibitor etomoxir that blocks fatty acid oxidation (FAO) (Fig. 4a, Extended Data Fig. 4a). To investigate directly whether RalA plays a role in controlling FAO, we incubated cells with (^14^C)-labeled palmitic acid (PA) and measured its oxidation to either acid-soluble metabolites (ASM) or CO_2_ in WT and KO white adipocytes. In agreement with the OCR results, fatty acid oxidation was significantly higher in KO compared to WT adipocytes (Fig. 4b). These data indicate that RalA knockout in WAT increases energy expenditure due to increased mitochondrial oxidation activity.

To ensure that these studies reflected the activity of RalA, we also generated an immortalized preadipocyte line from *Rala*^f/f^ mice and induced *Rala* deletion by transducing cells with Ore lentivirus. The Ore recombinase completely ablated RalA in preadipocytes and fully differentiated adipocytes (Extended Data Fig. 4b). BODIPY staining demonstrated that both primary and immortalized preadipocytes from WT and KO mice were fully differentiated. As an orthogonal approach, we performed live cell imaging using the cell permeant fluorescent dye, TMRM, to detect mitochondrial membrane potential (MtMP), which reflects electron transport and oxidative phosphorylation in active mitochondria. KO adipocytes exhibited a higher TMRM signal intensity than did their WT counterparts (Fig. 4c, Extended Data Fig. 4c). To specify the ability of TMRM to detect mitochondrial depolarization in active mitochondria, we applied the β3-adrenergic receptor agonist CL316,243 (CL) to induce mitochondrial membrane depolarization ^29^. The TMRM signal declined quickly after administration of the agonist, which confirms that TMRM stains only active mitochondria (Extended Data Fig. 4d).

We previously reported that lipolysis drives mitochondrial oxidative metabolism in adipocytes ^30^. To rule out a possible role for lipolysis as the primary driver of increased oxidative capacity of *Rala*-KO adipocytes, we performed *in vitro* and *in vivo* lipolysis assays. CL robustly stimulated FFA and glycerol release to the same extent in KO and WT immortalized adipocytes, and the molar ratio of FFA to glycerol was approximately 3:1 (Extended Data Fig. 4e,f). Additionally, there was no difference in CL-induced FFA and free glycerol production in *Rala*^f/f^ and *Rala*^AKO^ mice (Extended Data Fig. 4g,h). We further tested whether *Rala*^AKO^ mice are defective in the suppression of FFA release by insulin. Insulin suppressed CL-induced FFA release by approximately 50% in both WT and KO cells (Extended Data Fig. 4e). A single injection of insulin reduced FFA levels in control and *Rala*^AKO^ mice to the same extent (Extended Data Fig. 4i). Interestingly, KO adipocytes displayed showed a mild decrease of plasma glycerol levels a mild increase in glycerol release in the presence of CL, while *Rala*^AKO^ mice either in the presence of CL or after fasting (Extended Data Fig. 4f,h,j). Taken together, these results suggest that the absence of RalA in adipocytes enhances mitochondrial oxidative activity without affecting FFA supply.

### Targeted Rala knockout protects against obesity-induced mitochondrial fission in iWAT

The increased mitochondrial oxidative activity observed in HFD-fed *Rala*^AKO^ mice could result from increased mitochondrial biogenesis. Expression of genes related to mitochondrial biogenesis was comparable between the genotypes (Extended Data Fig. 5a,b) in WAT. The activity of AMPK, the master regulator of mitochondrial biogenesis ^31,32^, was also comparable between control and *Rala*^AKO^ mice fed with HFD (Extended Data Fig. 5c-f). In addition to biogenesis, mitochondrial function can also be regulated by dynamic changes in morphology through tightly controlled fusion and fission events that shape the organelle to comply with energy demands ^19,33^. Electron microscopy (EM) revealed that HFD feeding of WT mice induced the appearance of smaller, spherical mitochondria in iWAT (Fig. 4d), consistent with previous reports that mitochondrial function and morphology is impaired in obese adipocytes ^34,35^. In agreement with the *In vivo* metabolic phenotypes, adipocyte *Rala* deletion did not grossly affect mitochondrial morphology in iWAT of CD-fed mice (Fig. 4d), but the HFD-induced change in mitochondrial morphology was completely prevented in *Rala* KO iWAT; mitochondria in iWAT from these mice displayed an elongated shape that was indistinguishable from CD-fed mice (Fig. 4d). Indeed, tissue weight (Fig. 1f), OXPHOS content (Extended Data Fig. 3o,p), and mitochondrial OCR (Fig. 3f) were not affected by RalA deletion in eWAT, corresponding to the observation that the appearance of fragmented mitochondria in this depot was not reversed by *RalA* KO in HFD mice (Extended Data Fig. 5g). In fact, mitochondria in eWAT do not undergo significant fragmentation in response to HFD, possibly because of their already fragmented shape, consistent with the overall anabolic function of visceral adipocytes (Fig. 4d, Extended Data Fig. 5g). We also examined mitochondrial morphology in immortalized adipocytes differentiated from iWAT. As shown in Fig. 4e, mitochondria in KO adipocytes appeared longer than those in WT cells. There was a higher frequency of elongated mitochondria (1.0–1.5 μm) in KO cells (Fig. 4f), and the mean maximal mitochondrial length was significantly higher than in WT cells (Fig. 4g).

## Inhibition of RalA increases Drp1 S637 phosphorylation in white adipocytes

**Figure 5:**
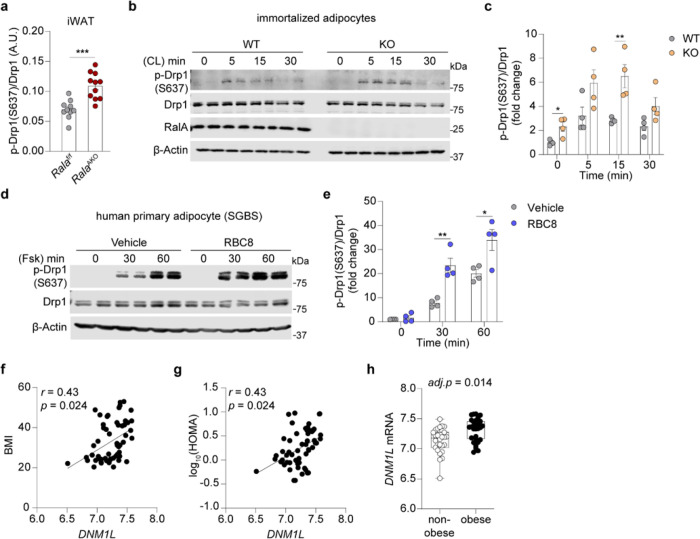
Inhibition of RalA increases Drp1 S637 phosphorylation in white adipocytes. **a,** Quantification of phospho-Drp1 (S637), total Drp1 and β-Tubulin immunoblotting in iWAT of HFD-fed mice (n = 10–13). **b, c,** Immunoblotting **(b)** and quantification **(c)** of phospho-Drp1 (S637), total Drp1, RalA, and (B-Actin in immortalized adipocytes (n = 4). Adipocytes were treated with 1 μM CL 316,243 (CL) for indicated time, **d, e,** Immunoblotting **(d)** and quantification **(e)** of phospho-Drp1 (S637), total Drp1 and β-Actin in human primary adipocytes (SGBS) (n = 4). Cells were pretreated with 50 μM RBC8 or DMSO for 30 min prior to treatment with 20 μM forskolin (Fsk) for indicated time, **f, g,** *DNM1L* mRNA expression is correlated with BMI **(f)** and HOMA **(g)** in human abdominal subcutaneous adipose tissue samples (n = 56). Significance in correlation was assessed by Spearman’s correlation test, **h,** Box-and-whisker plot of *DNM1L* mRNA expression in abdominal subcutaneous adipose tissues from non-obese and obese human subjects (n = 56). The data **(a, c, e)** are shown as the mean ± SEM, *p < 0.05, **p < 0.01, ***p < 0.001 by unpaired t-test **(c,e).**

Opa1 and Drp1 have been identified as key regulators of mitochondrial fusion and fission, respectively ^36^. Opa1 undergoes proteolytic cleavage to generate long (L-Opa1) and short (S-Opa1) forms that together fuel mitochondrial fusion ^37,38 39^. Protein levels of both forms of Opa1 were downregulated in iWAT after HFD feeding (Extended Data Fig. 5h-j); only S-0pa1 was downregulated in eWAT from *Rala*^AKO^ mice (Extended Data Fig. 5k-m), indicating the likelihood of reduced fusion in KO mice compared to WT littermates. However, the observation of elongated mitochondria in KO mice (Fig. 4d) suggests that this change in Opa1 processing is likely compensatory. We then focused on Drp1 as a key regulator of fission. Interestingly, Drp1 phosphorylation at the anti-fission S637 site was significantly increased in *Rala-KO* iWAT (Fig. 5a, Extended Data Fig. 6a), whereas Drp1 S637 phosphorylation was comparable between the genotypes in eWAT (Extended Data Fig. 6b,c). To establish whether this effect is cell-autonomous, we examined Drp1 phosphorylation in both immortalized and primary adipocytes. Drp1 S637 phosphorylation is catalyzed by PKA, activated by the b-adrenergic/cAMP pathway ^40,41^. Drp1 S637 phosphorylation was triggered by CL after 5 minutes and was maximal after 15 minutes in adipocytes (Fig. 5b, Extended Data Fig. 6d). Consistent with *in vivo* results, *Rala-KO* adipocytes showed a significantly higher Drp1 S637 after β-adrenergic stimulation compared to WT cells (Fig. 5c, Extended Data Fig. 6e). We also explored the effect of RalAon Drp1 S637 phosphorylation state using a specific Ral inhibitor that prevents activation and retains the GTPase in the GDP-bound, inactive state. Pretreatment with the pan-Ral inhibitor RBC8 ^26,42^ significantly increased forskolin-stimulated Drp1 S637 phosphorylation in 3T3-L1 adipocytes (Extended Data Fig. 6f,g). Inhibition of RalA activity with RBC8 also increased forskolin-stimulated Drp1 S637 phosphorylation in the human primary adipocyte cell line (SGBS) (Fig. 5d,e). Thus, RalA specifically modulates Drp1 S637 phosphorylation downstream of PKA activation across multiple adipocyte cell lines of both murine and human origin. To determine whether RalA influences CL-induced PKA activation or cAMP breakdown, we measured cAMP production and phosphorylation of hormone sensitive lipase (HSL) in adipocytes. There was no difference in cAMP production between WT and KO primary adipocytes after 5 minutes of CL stimulation (Extended Data Fig. 6h). Similarly, HSL S660 phosphorylation was identical in WT and KO adipocytes (Extended Data Fig. 6i-l).

To examine the relevance of Drp1 as a regulator of metabolism in human obesity, we analyzed microarray data of abdominal subcutaneous WAT from obese and non-obese women. In human subcutaneous WAT, *DNM1L* (encoding human Drp1 protein) expression was positively correlated with BMI and HOMA (Fig. 5f,g), and its expression was significantly upregulated in obese subjects (Fig. 5h), indicating that increased expression of *DNM1L* may contribute to mitochondrial dysfunction in obesity. Moreover, bioinformatic analysis of published microarray data (GEO: GSE7053) from 770 human males further confirmed that *DNML1* is associated with obesity (Extended Data Fig. 6m-o). Together, these *in vivo and in vitro* data suggest that upregulated Drp1 activity in adipose tissue may be an important contributor to mitochondrial dysfunction during obesity and further that RalA deficiency protects mitochondria from excessive fission by increasing Drp1 S637 phosphorylation.

## RalA interacts with Drp1 and protein phosphatase 2A, promoting dephosphorylation of Drp1 at S637

**Figure 6:**
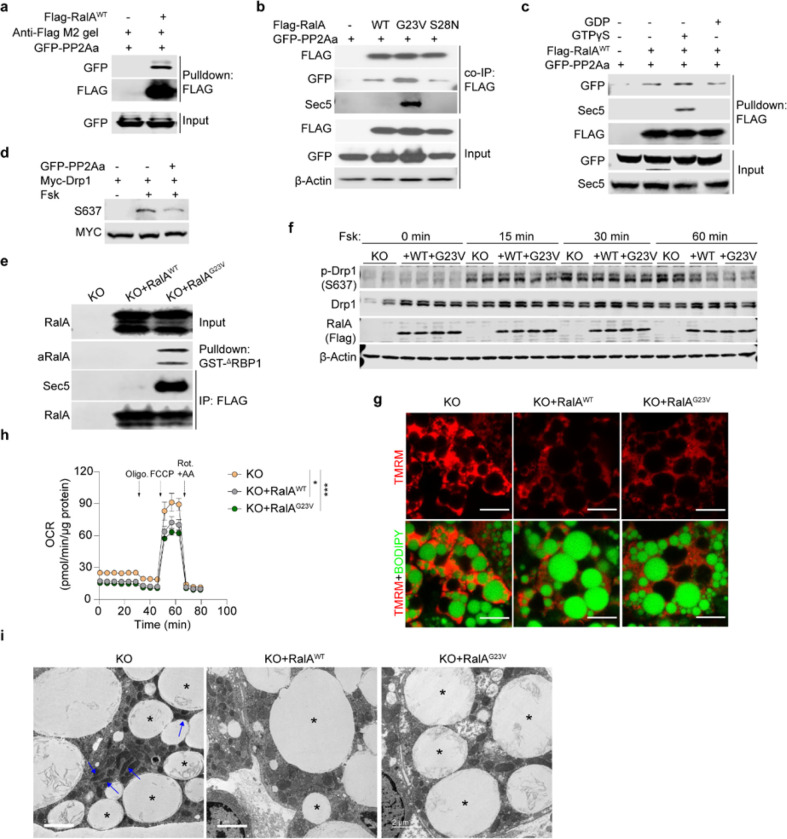
RalA interacts with Drp1 and protein phosphatase 2A, promoting dephosphorylation of Drp1 at S637. **a,** Pull down assay determining PP2Aa-RalA interaction. Purified Flag-RalA^**WT**^ was used to pull down GFP-PP2Aa overexpressed in HEK293T cells, **b,** Immunoblot analysis of co-immunoprecipitation determining interaction between RalA wildtype (WT), constitutive active (G23V), or dominant negative (S28N) mutants and PP2Aa in HEK293T cells, **c,** Flag-pull down assay determining interaction between PP2Aa and GTP/GDP-loaded RalA. Purified Flag-RalA^**WT**^ protein loaded with either GTPγS or GDP was respectively used as a prey to pull down GFP-PP2Aa from HEK293T cells, **d,** *In vitro* dephosphorylation assay in HEK293T cells co-transfected with PP2A and Drp1 plasmids. Cells were treated for 1 hr with 20 μM forskolin (Fsk) or vehicle, **e,** RalA activity assay in immortalized *RalA* KO adipocytes reconstituted with RalA^**WT**^ and RalA^**G23V**^. **f,** Immunoblottmg of phospho-Drp1 (S637), total Drp1, Flag-tagged RalA and β-Actin in immortalized *RalA* KO adipocytes with or without RalA reconstitution (n = 3). Adipocytes were treated with 20 μM forskolin for indicated time, **g,** Representative confocal images of live immortalized adipocytes (n = 3) stained with TMRM (red) and BODIPY (green), scale bar = 15 μM. **h,** OCR was measured by seahorse in immortalized adipocytes (n = 5–6). Vertical arrows indicate injection ports of indicated chemicals. Data are shown as the mean ± SEM, *p < 0.05, ***p < 0.001 by two-way ANOVA. **i,** Representative EM images (n = 3) of *RalA* KO immortalized adipocytes with or without RalA reconstitution. Blue arrow indicates elongated mitochondria; asterisk indicates lipid droplet. Scale bar = 2 μm.

To understand the molecular mechanism by which RalA regulates Drp1 S637 phosphorylation, we used proteomics to search for proteins interacting with wildtype (WT), constitutively active (G23V), or dominant negative (S28N) forms of RalA ectopically expressed in liver. Among the binding proteins was protein phosphatase 2A subunit A alpha (PP2Aa), the scaffolding subunit encoded by the *Ppp2r1a* gene, which preferentially bound to the RalA^G23V^ constitutively active mutant. To confirm these mass spectrometry data, we purified RalA^WT^-Flag protein from HEK293T cells and pulled down PP2Aa from lysates (Fig. 6a). To determine whether this interaction is dependent on the activation state of the G protein, we co-expressed WT and mutant RalA constructs with PP2Aa in FIEK293T cells. As a positive control, the effector Sec5 only bound to active RalA^G23V^. Similarly, this mutant form of RalA had the highest affinity for PP2Aa (Fig. 6b). We also loaded a RalA-Flag fusion protein *in vitro* with GTPγS or GDP to evaluate the specificity of effector binding. Both Sec5 and PP2Aa were pulled down by RalA loaded with GTPγS but not with GDP (Fig. 6c). In addition, because PP2Aa and Drp1 did not independently interact (data not shown), we investigated whether RalA directly modifies Drp1 phosphorylation via PP2Aa. When co-expressed, Drp1 and RalA interacted directly with each other, although there was no preference for the activation state of RalA (Extended Data Fig. 7a). Activation of the cAMP/PKA axis by addition of forskolin increased Drp1 S637 phosphorylation, while co-expression of PP2Aa promoted the dephosphorylation of S637 (Fig. 6d), although overexpression of PP2Ab had no effect (Extended Data Fig. 7b). These data suggest that Drp1 is constitutively associated with RalA independent of activation state, and upon activation, RalA recruits PP2Aa to promote the dephosphorylation of Drp1 S637.

To understand further the effects of RalA activation state on Drp1 phosphorylation and mitochondrial function, we transduced immortalized RalA KO cells with RalA^WT^ and RalA^G23V^ lentivirus prior to differentiation into adipocytes. RalA^G23V^ expressing adipocytes showed a robust increase in RalA GTP binding (Fig. 6e), and these cells had significantly less Drp1 S637 phosphorylation (Fig. 6f, Extended Data Fig. 7c). Expression of either RalA^WT^ or RalA^G23V^ significantly reduced mitochondrial potential in KO adipocytes (Fig. 6g, Extended Data Fig. 7d). To confirm that this reduction in mitochondrial potential is associated with reduced oxidative function, we performed a seahorse assay. Consistent with results in primary adipocytes, RalA^WT^ and RalA^G23V^ expressing adipocytes displayed reduced basal and maximal oxygen consumption rate (OCR) in comparison to KO adipocytes (Fig. 6h, Extended Data Fig. 7e). In addition, EM revealed that overexpression of WT or constitutively active RalA in adipocytes resulted in fragmented mitochondria, indicating increased fission compared to RalA KO adipocytes (Fig. 6i).

RalA has previously been reported to promote fission in proliferating cells, and *Rala* knockdown led to a long, interconnected mitochondrial network and reduced proliferation ^43^. Partially in agreement with this study, we found that RalA deficiency resulted in elongated mitochondria in adipocytes, with increased oxidative phosphorylation that dramatically impacted whole body lipid metabolism. However, unlike the previous study, we did not observe an interaction between RalBPI and Drp1. Interestingly, total PP2Aa protein levels were increased in *Rala* KO iWAT compared to control iWAT, without a difference in PP2Ab and PP2Ac content (Extended Data Fig. 7f,g), perhaps reflecting a compensatory pathway. Taken together, our data suggest that obesity drives RalA expression and GTP binding activity, leading to its association with PP2Aa, which in turn recruits the catalytic subunit PP2Ac to dephosphorylate Drp1 S637. We also note that catecholamine resistance, an inherent trait of the obese state ^28^, is also expected to lead to reduced PKA-catalyzed S637 phosphorylation. Together, these effects result in constitutive mitochondrial translocation of Drp1 and fragmented mitochondria in adipocytes from obese subjects (Extended Data Fig. 8).

## Discussion

While accumulating evidence suggests that mitochondrial dysfunction is a characteristic trait of obesity in human and rodent adipocytes ^16,34,35,44^, the underlying molecular mechanisms remain unknown. Here, we report a new regulatory axis for the control of mitochondrial morphology and function in the context of obesity involving prolonged activation of the small GTPase RalA. We show that RalA is both induced and activated in white adipocytes after feeding rodents a high fat diet, while the negative regulator of RalA, RalGAP is down regulated. We also observe a positive correlation of expression of the RalGEF RGL2 with BMI in adipose tissue of humans with obesity, expected to correspond to a chronic increase in RalA activity. The increase in adipocyte RalA mRNA, protein, and activity is associated with mitochondrial dysfunction, characterized by fragmentation and reduced oxidative capacity, specifically in iWAT. Targeted deletion of RalA in white adipocytes prevents the obesity-dependent fragmentation of mitochondria and produces mice resistant to HFD-induced weight gain via increased energy expenditure. *In vitro* studies revealed that RalA suppresses mitochondrial oxidative function in adipocytes by increasing fission through reversing the inhibitory phosphorylation of the mitochondrial fission protein Drp1. This reduced phosphorylation results from the recruitment of the regulatory subunit of PP2A, which acts as a *bona fide* effector of RalA, leading to the specific dephosphorylation of the inhibitory Ser^637^ residue on Drp1, rendering the protein active. We also note our previous study in which constitutive activation of RalA via adipocyte-specific KO of *Ralgapb* produced a significant enlargement of white adipocytes and increased adipose tissue mass, even on a control diet ^26^. Thus, chronic elevation in RalA activity plays a key role in repressing energy expenditure in obese adipose tissue, contributing to weight gain and related metabolic dysfunction, including glucose intolerance and fatty liver, and may explain in part how energy expenditure is repressed in prolonged obesity ^45^.

The observation that adipocyte RalA controls overall systemic metabolism via this mechanism was surprising. We and others previously reported that RalA plays a key role in controlling the trafficking of GLUT4 vesicles in adipocytes and muscle ^22,23^. RalA is activated by insulin, mainly by inhibition of its GAP complex through phosphorylation ^24,46^, and when activated, RalA interacts with components of the exocyst complex to target GLUT4 vesicles to the plasma membrane for fusion, increasing glucose uptake into fat cells ^21^. Indeed, adipocytes treated with a RalA inhibitor ^26^ or isolated from RalA KO mice showed dramatically reduced GLUT4 translocation to the plasma membrane, with less glucose uptake in response to insulin. Targeted deletion of the scaffolding subunit of the RalGAP complex resulted in constitutive activation of RalA in adipocytes and myocytes, and dramatically improved glucose homeostasis ^22,26,46^. However, detailed physiological tracer studies revealed that improvements in glucose disposal in adipocyte-specific KO mice occurred primarily in brown fat, where glucose uptake was markedly increased ^26^. Consistent with these findings, we observed a small but significant reduction in insulin sensitivity in *Rala*^AKO^ mice on control diet, accompanied by reduced weights of all adipose tissues, likely reflecting less nutrient uptake. However, *Rala*^AKO^ mice on HFD paradoxically showed improved glucose tolerance and insulin sensitivity. While it remains unclear exactly how these mice overcome the negative effects of RalA deletion on glucose uptake, GLUT4 mRNA and protein levels in WAT are downregulated in obesity ^12,47,48^ GLUT1 mRNA and protein levels are increased ^49,50^, consistent with our RalGAP KO studies in HFD-fed mice that show little glucose uptake into white fat in response to insulin, but higher basal levels ^26^. Thus, it seems likely that improved glucose tolerance in *Rala*^AKO^ mice occurs because of weight loss and increased energy expenditure.

It was also interesting that liver function was dramatically improved in *Rala*^AKO^ mice on HFD, with reduced hepatic lipids and gluconeogenesis, as indicated by improvements in pyruvate tolerance. It is well established that WAT plays an important role in regulating whole-body energy metabolism ^51^. Hepatic acetyl-CoA arises from WAT lipolysis to directly promote hepatic gluconeogenesis ^52^. The increase in fatty acid oxidation in *Rala*-KO adipocytes resulted in less circulating FFAs and TGs, likely producing improved liver health and reduced gluconeogenesis.

While the significance of the adipose depot specificity of the effects of RalA remains uncertain, we note that adipocytes in visceral, subcutaneous, and brown fat differ in many ways ^53,54^. Although RalA was deleted in all adipocytes in *Rala*^AKO^ mice, mitochondrial function was only improved in iWAT. While there are numerous differences between visceral and inguinal white adipocytes that might explain this, including their response to HFD, one notable issue has to do with inherent mitochondrial morphology. Upon HFD feeding, adipocytes in iWAT underwent a dramatic size expansion, accompanied by a change in mitochondria from an elongated to a fragmented morphology, reflecting a transition to a largely anabolic state. These changes were not observed in RalA KO mice. Unlike what was observed in iWAT, mitochondria in eWAT display a fragmented morphology even in lean mice, with no change observed after HFD or RalA KO, consistent with the overall energy storage function of this depot even without the anabolic pressure of overnutrition.

Another question concerns the role of RalA in BAT. While BAT tissue weight was reduced in both *Rala*^AKO^ and *Rala*^BKO^ mice compared to controls, likely due to reduced glucose uptake, only iWAT adipocytes appear to respond with a change in metabolic activity and mitochondrial morphology. Brown adipocyte mitochondria are morphologically different from those in white adipocytes. These brown adipocyte mitochondria are more numerous and larger than the mitochondria in white adipocytes and contain packed cristae. Comparison of the mitochondria of brown and white adipocytes by proteomic analysis revealed that proteins involved in pathways related to fatty acid metabolism, OXPHOS, and the TCA cycle were highly expressed in BAT compared to WAT ^55^. Thus, it seems likely that mitochondria in BAT are subjected to fundamentally different modes of regulation than those in white fat, and the reduced weight of BAT in KO mice can be attributed to reduced glucose uptake.

As mitochondrial function is vital for healthy metabolism, efforts have focused on preventing fragmentation via blocking activity or direct deletion of Drp1 ^56^. Muscle mitochondrial dysfunction is closely related to excessive Drp1 activity ^57,^ and elevated Drp1 activating S616 phosphorylation has been found in severely obese human muscle ^58,59^. On the other hand, triggering Drp1 S637 phosphorylation has been suggested to increase the uncoupling capacity of FFA in brown adipocytes ^29^. In line with this observation, increased S637 phosphorylation was found in BAT after cold exposure ^60^. Administration of a Drp1 inhibitor acutely improved muscle insulin sensitivity and systemic glucose tolerance ^61,62^. However, the impact of modulating Drp1 levels is complicated and varies between tissues. Targeted deletion of Drp1 in liver reduced hepatic lipid accumulation and body weight in a NAFLD model ^63^. Moreover, loss of Drp1 impairs brown adipocyte differentiation and thermogenesis, possibly reflecting the aspects of mitochondrial morphology that are unique to BAT ^60,64^. Interestingly, ER stress has been observed in both tissue-specific Drp1 knockout mice models, which suggests that Drp1 may also regulate ER remodeling ^65^. These findings highlight the likely differences between total ablation of Drp1 activity and changes in its upstream regulatory pathways.

Many questions remain concerning the role of the RalA/Drp1 axis in the control of mitochondrial function in subcutaneous adipocytes. What is the mechanism by which RalA mRNA and protein expression are increased and RalGAP is decreased in adipose tissue during obesity? Additionally, the factors leading to increased RalA GTP binding are not known, although this may be a result of reduced RalGAP expression, as well as hyperinsulinemia and chronic elevations in Akt activity seen in obesity ^66^. A key question concerns the spatial compartmentalization of RalA activation and Drp1 phosphorylation/dephosphorylation in adipocytes. Are there pools of RalA in different cellular compartments that interact with different effectors? Do other isoforms of RalA (RalB) also control Drp1 localization and function? What is the domain of PP2Aa that interacts with RalA? While many questions remain, these findings open a new line of investigation concerning how the RalA/Drp1 axis regulates energy homeostasis.

## Methods

### Materials

#### Animals

RalA-floxed (*Rala*^f/f^) mice were bred with Adiponectin-promoter driven Cre or Ucp1-promoter driven Cre transgenic mice to generate fat depot specific RalA knockout (*Rala*^AKO^ or *Rala*^BKO^) mice. All mice have a C57BL/6J background, and all experiments were done using littermates. Male mice were used for *in vivo* experiments, and female mice were only used for primary preadipocyte isolation. We fed mice with standard chow diet (CD) (Teklad, #7912) or high fat diet (HFD) consisting of 60% calories from fat (Research Diets, #D12492) for 8–12 weeks, starting from 8 weeks old. Mice were housed in a specific pathogen-free facility with a 12-hr light and 12-hr dark cycle and given free access to food and water. All animal experiments were approved by and followed the guidelines from the Institutional Animal Care and Use Committee (IACUC) at the University of California, San Diego.

#### Cell lines

##### Primary preadipocytes.

The isolation of primary preadipocytes was done as described previously ^67^ with some modification. IWAT from two to three 8-week-old female mice was dissected, minced, and digested in 5 mL 1 mg/mL collagenase (Sigma) for 15 minutes (min) in a 37°C water bath with gentle agitation. DMEM/F12 medium (15 mM HEPES) with 10% fetal bovine serum (FBS) was added to stop digestion and cells were filtered through 100 μm and 70 μm strainers. After centrifugation, cells were plated onto one 10 cm dish and cultured in DMEM/F12-FBS medium. 3 days post isolation, cells were washed three times with DPBS to remove dead cells and cultured in fresh DMEM/F12-FBS medium. Once cells reached 90% confluence, preadipocytes were seeded into 12-well plates or imaging dishes for differentiation. Differentiation of primary preadipocytes was described elsewhere ^68^.

##### Immortalized adipocytes.

Primary preadipocytes from *Rala*^f/f^ mice were immortalized as previously described by retroviral transduction of pBabe-zeo-LT-ST(SV40) and selection by Zeocin ^69^. Single cell clones were selected and tested for differentiation capacity. All used clones in this study displayed 100% adipocyte morphology after differentiation. To generate *Rala* knockout (KO) cells, immortalized *Rala*^f/f^ (WT) preadipocytes were transduced with lentiviral Ore with 8 μg/mL polybrene for 12 hours (hrs), then cultured in DMEM/F12-FBS medium. Cre recombinase efficiency was tested in preadipocytes and adipocytes. Once reaching 95–100% confluence (Day 0), immortalized preadipocytes were induced in DMEM/F12-FBS medium containing 0.5 μM IBMX, 5 μM dexamethasone, 1 μM rosiglitazone and 5 μg/mL insulin for 3 days. Medium was then switched to DMEM/F12-FBS medium with rosiglitazone and insulin. On Day 5, cells were changed to insulin only DMEM/F12-FBS medium. On Day 7, cells were maintained in DMEM/F12-FBS medium until 100% differentiated. On the day of the experiment, cells were starved in DMEM/F12 medium for 3 hrs prior to adding treatments.

##### Human primary preadipocytes (SGBS).

Human SGBS cells were cultured as previously described ^70^, and differentiated with a published protocol ^71^.

##### 3T3-L1 adipocytes.

Murine 3T3-L1 fibroblasts were cultured in high glucose Dulbecco’s Modified Eagle’s Media (DMEM) with 10% newborn calf serum (NBCS). 2 days after reaching 100% confluence, cells were induced for differentiation in DMEM with 10% FBS (DMEM-FBS) medium supplied with 0.5 mM IBMX, 1.25 μM dexamethasone, and 2 μg/mL insulin for 3 days. Afterwards, cells were cultured in DMEM-FBS medium with 2 μg/mL insulin for another 2 days. After 48 hrs, cells were cultured in DMEM-FBS medium until fully differentiated. Only cultures in which over 95% of cells displayed adipocyte morphology were used for experiments.

##### HEK 293T cells.

HEK 293T cells were cultured in high glucose DMEM-FBS medium. On the same day as seeding, when cells reached 50% confluence, transfection was done as designed using lipofectamine 2000 (Life Technology) following the manufacturer’s protocol. Fresh DMEM-FBS medium was added 12–16 hrs after transfection. 48 hrs after transfection, cells reached around 80% confluency and were used for co-immunoprecipitation or pulldown experiments.

##### Lenti-X 293T cells.

Lenti-X 293T cells were cultured in high glucose DMEM-FBS medium for packing lentivirus. When cells reached 100% confluence on a 0.01% poly-lysine coated dish, 3rd generation lentiviral packaging plasmids (pLVX vectors, pMDLg/pRRE (Addgene#12251), pRSV-Rev (Addgene#12253) and pMD2.G(Addgene#12259)) were transfected into cells using lipofectamine 3000 (Life Technology) following the manufacturer’s protocol. Fresh DMEM-FBS medium with 25 mM HEPES was added 12–16 hrs after transfection. The lentivirus-containing medium was collected twice at 48 and 72 hrs post transfection. After collection, the medium was spun at 1,200 x rpm for 5 min to remove dead cells, then incubated with Lenti-X concentrator (Takara) with a 3:1 ratio at 4°C overnight. The viral pellets were collected by centrifugation at 1,500 x g for 45 min at 4°C and reconstituted in DMEM/F12-FBS medium with 8 μg/mL polybrene. Lentivirus was added to cells immediately after reconstitution.

##### Reconstitution of RalA ^WT^and RalA ^G23V^in RalA KO preadipocytes.

Immortalized RalA KO preadipocytes were transduced with concentrated Flag-RalA^WT^ or Flag-RalA^G23V^ lentiviral supernatants with 8 μg/mL polybrene. 24 hrs after infection, medium was changed to fresh DMEM/F12-FBS and expanded for differentiation. Expression of Flag-tagged protein was examined in fully differentiated cells by western blot.

### Gene analysis in clinical cohorts

The transcriptomics data from abdominal subcutaneous WAT of 30 obese and 26 healthy non-obese women were generated as previously described ^72^. Transcriptome profiles were obtained using GeneChip Human Gene 1.0 ST Arrays. Data were deposited in NCBI gene expression omnibus (GEO) with the accession number GSE25402. Transcriptome profiles in verification cohort were obtained from subcutaneous fat biopsies from 770 men participating in the METSIM study ^73^. Transcriptomics and clinical data were retrieved from GEO (GSE70353). Human subjects with BMI >30 kg/m^2^ are considered obese in these analyses.

### Primary mature adipocyte isolation

Minced white adipose tissue was digested in DMEM with 1 mg/ml collagenase (Sigma) for 25 min at 37°C with gentle agitation. The cell suspension was filtered through a 100 μm cell strainer and centrifuged at 50 x g for 3 min to separate floating mature adipocytes. Floating mature adipocytes were transferred to PBS with broad open tips and washed twice. 1 mL mature adipocytes were lysed in 4 mL TRIzol^™^ (Life Technology) for RNA isolation.

### RNA sequencing analysis

RNA extractions from primary mature inguinal and epididymal adipocytes were performed using TRIzol^™^ (Life technologies) and PureLink RNA mini kit (Life technologies), according to the manufacturer’s instructions. RNA quality was checked by Agilent TapeStation. Biological triplicates of isolated 500 ng RNA were used to prepare sequencing libraries using the TruSeq RNA Sample Preparation Kit v2 (Illumina), according to the manufacturer’s protocol. Libraries were validated using a 2100 BioAnalyzer (Agilent), then normalized and pooled for sequencing using bar-coded multiplexing at a 90-bp single-end read length on an Illumina HiSeq 4000. Samples were sequenced to a median depth of 14 million reads.

### Bioinformatics analysis

For RNA-Seq, sequencing fastq files were generated automatically using the Illumina bcl2fastq2 Conversion Software. Read alignment and junction mapping to genome mm39 (GRCm39) and the mouse Genecode M30 annotation were accomplished using STAR (version 2.7.2b). Known splice junctions from mm10 were supplied to the aligner and de novo junction discovery was also permitted. Differential gene expression analysis and statistical testing were performed using DESeq2. Differentially expressed genes were defined as having an adjusted *P* value < 0.05. Raw gene counts were normalized to FPM (fragments per million mapped fragments) using DEseq2. FPM counts were filtered, centered by z-score before gene clustering and heatmap generation using GENE-E (v3.0.215) or GraphPad Prism (8.4.3). For microarray data, gene matrix files were collapsed by Collapse Dataset tool in GSEA (4.3.2) using chip platform (GPL13667) with collapsing mode (Mean_of_probes). Statistical significance of differential gene expression was assessed by ComparativeMarkerSelection module (version 11) from GenePattern (https://cloud.genepattern.org/gp/pages/index.jsf).

### Gene expression analysis

Tissue RNA was isolated with TRIzol^™^ reagent in combination with column (PureLink RNA mini, Invitrogen) according to the manufacturer’s protocol. cDNA was generated from 1 μg RNA using the cDNA Maxima Reverse Transcription Kit (Thermo Fisher Scientific). mRNA expression was assessed by real-time PCR using QuantStudio 5 real-time PCR system and SYBR Green PCR master mix (Invitrogen). Gene expression was normalized to *Cyclophilin A* in murine tissues. Relative mRNA expression levels were calculated using averaged 2^−ΔΔCt^ values for each biological replicate. Primers are listed in Extended Data Table 1.

### Protein isolation and western blotting

Tissue or cells were lysed or homogenized in RIPA buffer with freshly added Halt^™^ Protease and Phosphatase Inhibitor cocktail (ThermoFisher). Lysates were rotated in a cold room for 30 min, then briefly sonicated and centrifuged at 17,000 x g for 15 min at 4°C. Cleared supernatants were collected, and concentrations were determined with BCA protein assay kit (Pierce) for quantification. Proteins were resolved by Tris-Glycine gel (Novex, Invitrogen) electrophoresis and transferred to nitrocellulose membranes. Individual proteins were detected with the specific antibodies (OXPHOS ab110413, β-Tubulin 2146S, phospho-Drp1 (Ser637) 4867S, phospho-HSL(Ser660) 45804S, HSL 4107S, MYC 2276S, Drp1 8570S, phosphor-AMPK(Thr172) 2535S, AMPK 5831S, RalA BD610221, β-Actin 66009-1-Ig, FLAG 66008-4-lg, GFP 66002-1-Ig, Sec5 12751-1-AP) and visualized on blots using fluorescent secondary antibodies (mouse 926-32210, rabbit 926-68071) with Li-Cor system or on film using horseradish peroxidase-conjugated secondary antibodies (Fisher Scientific) with SuperSignal West Pico Chemiluminescent substrate (ThermoFisher). Bands were quantified with ImageStudio or ImageJ.

### Body mass composition

Body mass composition was assessed in non-anesthetized mice by using EchoMRI.

### Glucose tolerance test

Mice were fasted for 6 hrs, then intraperitoneally (i.p.) injected with D-[+]-glucose in PBS at a dose of 2 g/kg BW for CD-fed mice or 1.2 g/kg BW for HFD-fed mice. Blood glucose levels were measured before injection and at 15, 30, 60, 90, and 120 min after injection using the Easy Touch glucose monitoring system.

### Insulin tolerance test

Mice were fasted for 4 hrs, then i.p. injected with human insulin (Sigma) in saline at a dose of 0.35 U/kg BW for CD-fed mice or 0.6 U/kg BW for HFD-fed mice. Blood glucose levels were measured before injection and at 15, 30, 60, 90, and 120 min after injection using the Easy Touch glucose monitoring system.

### Pyruvate tolerance test

Mice were fasted for 16 hrs, then i.p. injected with pyruvate in PBS at a dose of 1.5 g/kg BW for HFD-fed mice. Blood glucose levels were measured before injection and at 15, 30, 60, 90, and 120 min after injection using the Easy Touch glucose monitoring system.

### Blood parameters

Whole blood was taken from the facial vein and blood glucose was measured with a glucose meter (Easy Touch) from the tail vein. Plasma was collected after centrifugation at 1,200 x rpm, 4°C for 10 min. Plasma triglyceride (TG) and free fatty acid (FFA) levels were measured with Infinity^™^ Triglycerides kit (Thermo Fisher) and NEFA kit (WAKO). Plasma insulin levels were measured with the Mouse Ultrasensitive Insulin ELISA kit (Crystal Chem, #90080), and leptin levels were measured with Mouse Leptin ELISA (Crystal Chem, #90030) kit. Plasma AST and ALT activity was measured with the Aspartate Aminotransferase Activity kit (Biovision, #K753) and Alanine Aminotransferase Activity kit (Biovision, #K752).

### HOMA-IR calculation

Homeostasis model assessment of insulin resistance (HOMA-IR) is an index of overall insulin sensitivity ^74^. Glucose and insulin levels from overnight fasted mice were measured as described above. The values were used to calculate HOMA-IR with the formula: fasting insulin (μU/L) x fasting glucose (nmol/L) / 22.5.

### Hepatic lipid TG measurement

Frozen liver tissue (50–100 mg) was homogenized in 1 mL of PBS. 800 uL of lysates were added to 4 mL extraction buffer. After thoroughly rotating for 30 min at room temperature, the lipid phase was separated from the aqueous phase by centrifuging at 3,000 x rpm for 20 min. 0.2 mL of lipid fraction in the organic phase was collected and transferred to a 1.5 mL tube to dry under nitrogen stream in the fume hood. 0.2 mL of 2% Triton X-100 solution was used to solubilize the lipids. Triglyceride levels were determined by using the Infinity^™^ Triglycerides kit (Thermo Fisher). Lipid amount was normalized to liver lysate protein amount.

### Histology

For H&E staining, liver tissue was harvested and fixed in 10% formalin. Paraffin-embedding, sectioning, and hematoxylin & eosin (H&E) staining was completed at the UC San Diego Biorepository and Tissue Technology Shared Resources (BTTSR). For adipocyte size quantification, H&E slides were imaged using Keyance brightfield microscope or a Nikon confocal microscope with Texas Red excitation and emission filter. Adipocyte size was assayed using Adiposoft in Image J and an in-house developed pipeline with Cell Profiler according to previous descriptions ^30^. For Oil-Red-O staining, liver tissue was fixed in 4% PFA at 4°C for 24 hrs, then transferred to 20% sucrose/PBS for 24 hrs. Afterwards, tissue was embedded in O.C.T. (Sakura) with dry ice and ethanol. Frozen tissue blocks were sectioned and stained with Oil-Red-O at the BTTSR at UCSD.

### Indirect calorimetric measurements

For metabolic cage study, mice were individually housed in Promethion metabolic cages maintained at 22°C under a 12-hr light/12-hr dark cycle. Prior to the experiment, mice were adapted to the metabolic cages for 2 days. The monitoring system records and calculates food intake, locomotor activity, oxygen consumption, carbon dioxide production, respiratory exchange ratio (RER), and energy expenditure (EE). Mice were provided with free access to water and food during the whole measurement. The data were exported with ExpeData software (SABLE SYSTEMS) and EE was analyzed using ANCOVA with body weight as a covariate by web-based CaIR tool ^75^.

### Respiration measurement

#### Intact Cells.

The cellular OCR was measured using an eXF96 Extracellular Flux Analyzer and analyzed by Agilent Seahorse Wave Software (Seahorse Bioscience). Prior to assay, 2,500 primary preadipocytes were seeded into XF96 microplates. Two days after reaching full confluency, adipogenic differentiation was initiated using a protocol mentioned above. Once fully differentiated, adipocyte culture medium was changed to assay medium containing 25 mM glucose, 1 mM pyruvate and 2 mM L-Glutamine and 0.5 mM carnitine without phenol red or sodium bicarbonate for 3 hrs. Prior to the measurement, cells were incubated in a CO_2_-free incubator for 15 min. Basal rates of respiration were measured in assay medium and followed with sequential injections of oligomycin (2 μM), FCCP (0.5 μM), and Rotenone with Antimycin A (each 0.5 μM). Oxygen consumption values were normalized to protein content.

#### Isolated mitochondrial.

Isolation of mitochondrial from HFD-fed mice, and the OCR with 2.5 ug isolated mitochondrial was performed as previously described ^30^.

### Fatty acid oxidation assay

Fatty acid oxidation (FAO) assay was modified from a previous described protocol ^76^. Fully differentiated primary adipocytes in 24-well plates were incubated in 0.5 mL DMEM per well containing 1 mM carnitine and 0.5 μCi/well [^14^C]-palmitic acid for 60 min at 37°C. Afterwards, 360 μL medium was collected and added to 40 μL 10% BSA in a 1.5 mL tube with a filter paper in the cap. 200 μL 1 M perchloric acid was added to the tube, and the cap was immediately closed tightly and incubated at room temperature. After 1 hr, captured CO_2_ and acid-soluble metabolites (ASM) were used to measure radioactivity. The cells were lysed in NaOH/SDS buffer (0.3 N/0.1%) to measure protein concentration. FAO rates were normalized to protein content.

### Confocal microscope imaging

#### Live cell.

Fully differentiated adipocytes were cultured in a glass bottom dish (Cellvis) and incubated in phenol red-free DMEM (imaging medium) with 100 nM TMRM (Thermo Fisher) for 30 min to indicate mitochondrial membrane potential, and BODIPY 493/503 (final 5 ug/mL, Life Technology) was added to label lipid droplets for the last 15 min. Cells were then washed three times with imaging medium. Live cell images were obtained with Nikon A1R confocal with 100 x or 60 x oil immersion objective. For time-lapse imaging, pictures were taken every 10 min.

#### Fixed cell.

Fully differentiated primary adipocytes were cultured in a glass bottom chamber (Lab-Tek). On the day of the experiment, cells were serum starved for 3 hrs and treated with 100 nM insulin. After 15 min, medium was removed and cells were fixed with ice-cold methanol and incubated at −20°C for 10 min. Cells were then washed twice with PBS and blocked with 10% goat serum in PBS with 0.1% Triton X-100 at room temperature for 30 min. After blocking, cells were incubated with primary antibody at 4°C overnight and secondary antibody for 1 hr at room temperature. Cells were washed three times with PBS before imaging with Nikon A1R confocal microscope using 10Ox oil immersion objective.

### Lipolysis

#### In vitro.

Fully differentiated primary adipocytes in a 24-well plate were serum starved in lipolysis medium (2% BSA-phenol red-free DMEM) for 3 hrs. For insulin treatment, 100 nM insulin was added to cells for 30 min starting at the 2.5th hour of starvation. After starvation, medium was replaced with 0.5 mL fresh lipolysis medium with vehicle, 1 μM CL, 100 nM insulin, or in combination. Medium was collected after 1 hr incubation at 37°C. Released free fatty acids and free glycerol levels were measured using 100 μL medium with NEFAkit (WAKO) and Free Glycerol Reagent (Sigma) according to the manufacturer’s protocol.

#### In vivo.

CD-fed mice were used for *in vivo* lipolysis. For CL-induced lipolysis, *ad libitum* fed mice were intraperitoneally (i.p.) injected with PBS or CL (1 mg/kg) for 60 min. Circulating free fatty acids and free glycerol levels were measured using 2 uL plasma with NEFA kit (WAKO) and Free Glycerol Reagent (Sigma). For insulin-suppressed lipolysis, overnight fasted mice were i.p. injected with insulin (0.5 U/kg) for 60 min. Circulating free fatty acids and free glycerol levels were measured at indicated conditions.

### Electron microscopy

#### Adipose tissue.

Dissected adipose tissue was immediately fixed with 2–3 drops of fixative buffer (2% paraformaldehyde, 2.5% glutaraldehyde in 0.15 M sodium cacodylate buffer pH 7.4). Fat tissues were gently removed and fixed at room temperature. After 2 hrs incubation, tissues were further cut into around 1 mm^3^ cubes and immersed in fixative buffer overnight at 4°C. Tissue cubes were postfixed in 1 % osmium 0.15 M sodium cacodylate buffer (SC buffer) for 1–2 hrs on ice, followed by five 10-min washes in 0.15 M SC buffer, then rinsed in ddH_2_O on ice. Washed tissues were stained with 2% uranyl acetate for 1–2 hrs at 4°C then dehydrated in an ethanol series (50%, 70%, 90%, 100%, 100%, 10min each time) and dried in acetone for 15 min at room temperature. Dried tissues were infiltrated with 50%:50% Acetone:Durcupan for 1 hr or longer at room temperature then changed to 100% Durcupan overnight. The next day, embedded tissues in Durcupan were placed in a 60°C oven for 36 to 48 hrs. Ultrathin sections (60 nm) were cut on a Leica microtome with a Diamond knife and then post-stained with both uranyl acetate and lead. Images were obtained by using a Jeol 1400 plus Transmission Electron Microscope equipped with a Gatan digital camera.

#### Immortalized cells.

Fully differentiated cells in a 6-well plate were quickly fixed with 2% glutaraldehyde in 0.1 M sodium cacodylate buffer (pH 7.4) at room temperature for 15 min then incubated at 4°C for 15 min. Afterwards, cells were scraped down and pelleted by centrifugation. Cell pellets were postfixed in 1% OsO4 in 0.1 M sodium cacodylate buffer for 1 hr on ice. The cells were stained all at once with 2% uranyl acetate for 1 hr on ice, then dehydrated in graded series of ethanol (50–100%) while remaining on ice. The cells were then subjected to one wash with 100% ethanol and two washes with acetone (10 min each) and embedded with Durcupan. Sections were cut at 60 nm on a Leica UCT ultra microtome and picked up on 300 mesh copper grids. Sections were post-stained with 2% uranyl acetate for 5 min and Sato’s lead stain for 1 min. Images were obtained by using a Jeol 1400 plus Transmission Electron Microscope equipped with a Gatan digital camera.

### cAMP measurement

To induce cAMP production, fully differentiated primary adipocytes were stimulated with 1 μM CL for 5 min. Cells were then immediately lysed in lysis buffer (0.1 N HCL) and cAMP levels were measured with the Direct cAMP Enzyme Immunoassay kit (Sigma) according to the manufacturer’s protocol.

### Pulldown and Co-immunoprecipitation

#### Active RalA pulldown.

Fully differentiated primary adipocytes or immortalized adipocytes were serum starved for 3 hrs in DMEM medium and treated with 100 nM insulin, if needed, for the indicated time. After two washes with ice-cold TBS, cells were lysed in RalA buffer (25 mM Tris, 130 mM NaCI, 10 mM MgCI_2_,10% glycerol, 0.5% NP-40, EDTA-free protease inhibitor) and lysates were incubated at 4°C for 15 min then cleared by centrifugation. Protein concentrations were measured with the DC protein assay (Bio-Rad) and 0.5–1 mg protein was used for incubation at 4°C with 20 μL GST^−Δ^RalBP1 agarose beads (Millipore) for 45 min or 20 pL ANTI-FLAG^™^ M2 Affinity gel (Sigma) overnight. After incubation, beads were washed three times with RalA buffer and boiled at 65°C in 2X SDS buffer for 10 min.

#### Pulldown.

HEK293T cells cultured in 15-cm dishes were transfected with Flag-RalA^WT^ or GFP-PP2Aa. 48 hrs after transfection, cells were washed twice with ice-cold TBS then lysed on ice with 1 mL lysis buffer (25 mM Tris-HCI, 130 mM NaCI, 10 mM MgCI_2_,10% glycerol, 0.5% NP-40, EDTA-free protease inhibitor). Cell lysates were rotated for 15 min at 4°C and cleared by centrifugation for 15 min at 17,000 x g at 4°C. Flag-RalA^WT^ lysates were incubated with 20 μL ANTI-FLAG^™^ M2 Affinity gel (Sigma) at 4°C. After 2 hrs rotation, the empty M2 or Flag-RalA^WT^ beads were washed three times with lysis buffer then incubated with GFP-PP2Aa lysates at 4°C overnight. The next day, beads were washed three times with washing buffer (25 mM Tris-HCI, 40 mM NaCI, 30 mM MgCI_2_, 0.5% NP-40, EDTA-free protease inhibitor) and boiled in 2X SDS buffer at 65°C for 10 min. For GTPγS and GDP loading to Flag-RalA^WT^ beads, washed beads were rinsed with loading buffer (20 mM Tris, 50 mM NaCI, 1 mM DTT, 2 mM EDTA) then incubated with 2 mM GTPγS or 200 μM GDP in loading buffer for 1 hr at 25°C with 600 x rpm agitation. After loading, 10 mM MgCI_2_ was added to stop the loading, and loaded beads were incubated with GFP-PP2Aa lysates as above.

#### Co-immunoprecipitation.

For the co-IP experiment, it is critical to harvest cells at around 70–80% confluency. Co-transfected cells were washed twice with ice-cold TBS and lysed in 0.5 mL lysis buffer (the same as above) or Drp1 buffer (25 mM Tris, 50 mM NaCI, 0.5 mM MgCI_2_,10% glycerol, 0.5% NP-40, EDTA-free protease inhibitor). Lysates were cleared by centrifugation and protein concentrations were measured with BCA (Pierce). 0.5–1 mg protein was used for incubation with 20 μL ANTI-FLAG^™^ M2 Affinity gel (Sigma) at 4°C. After overnight gentle rotation, beads were washed three times with washing buffer (the same as above) or Drp1 wash buffer (25 mM Tris, 50 mM NaCI, 0.5 mM MgCI_2_, 0.1 % NP-40, EDTA-free protease inhibitor) and boiled in 2X SDS buffer at 65°C for 10 min.

### Vector construction

pMIG-PP2Aa (#10884), pMIG-PP2Ab (#13804), and pcDNA3.1-Drp1 (#34706) plasmids were purchased from Addgene and subcloned into mEGFP-C1 (#54759) and pCMV-Myc-3B vectors. RalA^WT^, RalA^G23V^, and RalA^S28N^ plasmids were subcloned into a pLVX vector with 3x Flag tag for lentiviral production.

### Statistics and reproducibility

Statistical analyses were performed using GraphPad Prism (8.4.3). All data in bar graphs are shown as mean ± SEM. N represents the number of biological replicates. All experiments were performed at least 3 times independently. For comparison between two groups, datasets were analyzed by Two-tailed unpaired Student’s *T*-test. For experiments with a two-factorial design, multiple comparisons were analyzed by two-way ANOVA to determine the statistical significance between groups based on one variable. Differences in EE were calculated with CaIR using ANCOVA with body weight as a covariate. The significance of the correlations between gene expression with BMI and HOMA values were calculated using Spearman’s correlation test. Values of *p <* 0.05 were considered as significantly different.

### Schematics

Schematics were prepared using Adobe Illustrator or Biorender.com with publication permissions.

## Data Availability

RNA-Seq data reported in this paper have been deposited in NCBI SRA database (BioProject PRJNA727566). Human study data were deposited in NCBI gene expression omnibus (GEO) with the accession number GSE25402 and retrieved from GEO (GSE70353).
